# Interictal SEEG Resting‐State Connectivity Localizes the Seizure Onset Zone and Predicts Seizure Outcome

**DOI:** 10.1002/advs.202200887

**Published:** 2022-05-12

**Authors:** Haiteng Jiang, Vasileios Kokkinos, Shuai Ye, Alexandra Urban, Anto Bagić, Mark Richardson, Bin He

**Affiliations:** ^1^ Department of Biomedical Engineering Carnegie Mellon University Pittsburgh PA 15213 USA; ^2^ Department of Neurobiology Affiliated Mental Health Center & Hangzhou Seventh People's Hospital Zhejiang University School of Medicine Hangzhou 310013 P. R. China; ^3^ NHC and CAMS Key Laboratory of Medical Neurobiology MOE Frontier Science Center for Brain Science and Brain‐machine Integration School of Brain Science and Brain Medicine Zhejiang University Hangzhou 310058 P. R. China; ^4^ University of Pittsburgh Comprehensive Epilepsy Center Department of Neurology University of Pittsburgh School of Medicine Pittsburgh PA 15232 USA; ^5^ Massachusetts General Hospital Boston MA 02114 USA; ^6^ Neuroscience Institute Carnegie Mellon University Pittsburgh PA 15213 USA

**Keywords:** connectivity, resting state, stereotactic‐electroencephalography (SEEG), seizure localization, seizure outcome, seizure‐onset zone

## Abstract

Localization of epileptogenic zone currently requires prolonged intracranial recordings to capture seizure, which may take days to weeks. The authors developed a novel method to identify the seizure onset zone (SOZ) and predict seizure outcome using short‐time resting‐state stereotacticelectroencephalography (SEEG) data. In a cohort of 27 drug‐resistant epilepsy patients, the authors estimated the information flow via directional connectivity and inferred the excitation‐inhibition ratio from the 1/*f* power slope. They hypothesized that the antagonism of information flow at multiple frequencies between SOZ and non‐SOZ underlying the relatively stable epilepsy resting state could be related to the disrupted excitation‐inhibition balance. They found flatter 1/*f* power slope in non‐SOZ regions compared to the SOZ, with dominant information flow from non‐SOZ to SOZ regions. Greater differences in resting‐state information flow between SOZ and non‐SOZ regions are associated with favorable seizure outcome. By integrating a balanced random forest model with resting‐state connectivity, their method localized the SOZ with an accuracy of 88% and predicted the seizure outcome with an accuracy of 92% using clinically determined SOZ. Overall, this study suggests that brief resting‐state SEEG data can significantly facilitate the identification of SOZ and may eventually predict seizure outcomes without requiring long‐term ictal recordings.

## Graphical abstract

This work presents a novel brain network analysis to identify the seizure onset zone and predict seizure outcome with an accuracy of around 90% using short‐time resting‐state stereotactic‐electroencephalography (SEEG) data, suggesting that brief resting‐state SEEG data has tremendous clinical values in diagnosis and aiding surgical planning in drug‐resistant epilepsy without requiring long‐term ictal recordings.

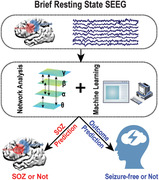

## 1 Introduction

Epilepsy is one of the most common neurological diseases^[^ ^1^ ^]^ impacting about 70 million people in the world. At least one‐third of epilepsy patients become drug‐resistant and potential candidates for surgical resection or neuromodulation treatment.^[^ ^2^ ^]^ The key to successful epilepsy surgery relies on accurate localization and safe removal of the epileptogenic zone (EZ)^[^ ^3^ ^]^ and an understanding of an individual patient's seizure network.^[^ ^4^ ^]^ An integral component for the delineation of the EZ is the seizure onset zone (SOZ): the area of cortex that initiates clinical seizures as determined predominantly by intracranial investigations.^[^ ^5^ ^]^ Although surgery and neuromodulation have been proven efficient in seizure reduction, the percentage of patients with unfavorable seizure outcomes leaves significant room for improvement.^[^ ^6^ ^]^

Stereotactic‐electroencephalography (SEEG) is a well‐established and safe neurosurgical approach^[^ ^7^ ^]^ to identify epileptic regions for intervention with intracerebral electrodes to record ictal/interictal brain activity.^[^ ^7^, ^8^ ^]^ The golden standard of localization of epileptogenic brain regions in clinical practice typically depends on capturing multiple seizures during the intracranial monitoring process, that may take multiple days or even weeks to complete.^[^ ^9^ ^]^ As such, a method which can estimate SOZ and predict prognosis outcome from analysis of brief, resting‐state data segments would have tremendous clinical values to identify epileptogenic networks without requiring prolonged intracranial recordings, which would vastly improve patient care and reduce medical cost.^[^ ^10^ ^]^

In a healthy state, the balanced excitation and inhibition in brain networks is regulated to facilitate information transfer and communications between remote functional regions.^[^ ^11^ ^]^ A number of studies have indicated that neuronal oscillations could transfer information at different frequencies, and oscillatory dysfunction has been implicated in almost every major psychiatric and neurological disorders.^[^ ^12^ ^]^ More specifically, it has been demonstrated that low‐frequency activity (LFA, <30 Hz), high‐frequency activity (HFA, >30 Hz), and LFA to HFA cross‐frequency interactions of the epilepsy network are disrupted.^[^ ^13^ ^]^ For example, high‐frequency oscillations (HFOs),^[^ ^14^ ^]^ interictal epileptiform discharges (IEDs),^[^ ^15^ ^]^ and phase‐amplitude coupling (PAC)^[^ ^16^ ^]^ have been widely investigated as promising clinical biomarkers for epilepsy.^[^ ^17^ ^]^ However, HFOs, IEDs, and PAC are all local biomarkers, while epilepsy is commonly considered as a network disease.^[^ ^18^ ^]^ The underpinnings of seizure generation involve abnormal brain structures and aberrant functional connections among these regions, leading to large‐scale network instability.^[^ ^19^ ^]^ Resting‐state network connectivity studies have suggested predominantly increased functional connectivity involving the EZ and surrounding structures,^[^ ^10a^ ^]^ and stronger inward directional connectivity toward EZ.^[^ ^10b^ ^]^ Furthermore, decreased interictal network synchrony and local heterogeneity were found to correlate with improved seizure outcome.^[^ ^20^ ^]^ Therefore, a better understanding of the functional architecture of the epileptic network could help identify SOZ and improve prediction of the seizure outcome.

**Figure 1:**
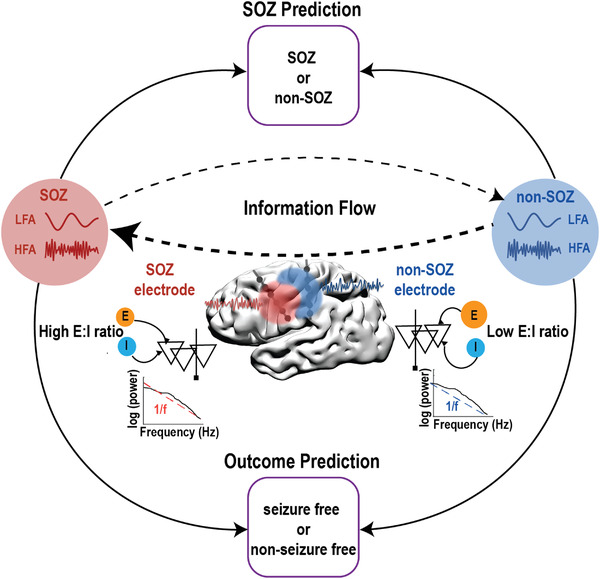
Schematic illustration of the study design. Within‐frequency and cross‐frequency directional connectivity (indication of information flow), 1/*f* power slope (indication of excitation and inhibition ratio) were investigated in the SEEG resting state data to predict SOZ and seizure outcome. LFA: low‐frequency activity; HFA: high‐frequency activity; SOZ: seizure‐onset zone; E:I: excitation:inhibition.

In this work, we investigate information flow in resting‐state epilepsy networks, inferred from directional connectivity in a cohort of 27 drug‐resistant focal epilepsy patients. We hypothesized that the excitation–inhibition balance is disrupted during epilepsy resting state compared to the healthy resting state and further reflected by aberrant information flow. Specifically, we hypothesized that during the relatively stable epilepsy resting state, there are antagonisms of information flow between SOZ and non‐SOZ regions at multiple frequencies. Furthermore, we speculated that the strength of antagonisms reflects intrinsic epileptic network characteristic, which is eventually associated with seizure outcome. The ultimate goal of this work is to develop a method to identify the SOZ for treatment intervention and to predict treatment outcomes, based on brief resting‐state SEEG data without necessitating prolonged ictal recordings (**Figure** **1**).

## 2 Results and Discussion

### 2.1 Dominant Information Flow from Non‐SOZ to SOZ Underlying the Resting State

**Figure 2:**
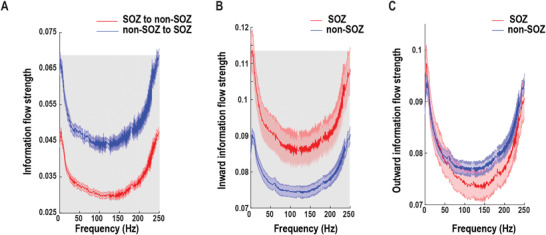
Within‐frequency information flow during the resting state. A) Mean bidirectional information flows between SOZ and non‐SOZ across all electrode pairs and patients. The shaded gray area indicates significant differences at the *p* = 0.01 level after multiple corrections. B) Inward (receiving) information flow strength in SOZ and non‐SOZ. The shaded gray area indicates significant differences at the *p* = 0.01 level after multiple corrections. C) Outward (sending) information flow strength in SOZ and non‐SOZ. Data are shown in mean and standard error.

**Figure 3:**
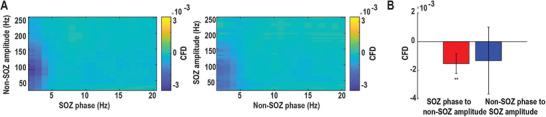
Cross‐frequency information flow during the resting state. A) Grand averaged SOZ phase to non‐SOZ amplitude CFD (left panel) and non‐SOZ phase to SOZ amplitude CFD (right panel) across all electrode pairs and patients. (B) Grand averaged CFD after the k‐means clustering procedure. The SOZ phase to non‐SOZ amplitude CFD is significant compared to zero. The error bar represents standard deviation. **p < 0.01.

First, we examined the confrontations of resting‐state information flows between SOZ and non‐SOZ by comparing their differences in directional interaction both within‐frequency and cross‐frequency. The within‐frequency directional information flow was calculated by directed transfer function (DTF),^[^ ^21^ ^]^ while the cross‐frequency directional information was estimated by cross‐frequency directionality (CFD).^[^ ^22^ ^]^ As shown in **Figure** **2**A, measures of within‐frequency information flow strength were significantly weaker from SOZ to non‐SOZ than in the other direction, over the wide frequency range (1–250 Hz). Furthermore, we observed that SOZ exhibited significantly higher inward strength (mean information received from other electrodes) than non‐SOZ (Figure 2B), but outward strength (mean information sent to other electrodes) did not differ between the regions (Figure 2C). Note that the patterns were reversed during the ictal period (Figure S1, Supporting Information). In the cross‐frequency directional interactions, both the SOZ phase to non‐SOZ amplitude and non‐SOZ phase to SOZ amplitude CFD showed prominent negative 1–4 to 40–150 Hz CFD (**Figure** **3**A), indicating information flow from HFA to LFA. Since CFD varied across electrode pairs to a different extent, we utilized k‐means clustering to extract the most consistent and strongest CFD pattern across all electrode pairs.^[^ ^23^ ^]^ After applying the k‐means procedure in each patient, we found the significant negative CFD from SOZ phase to non‐SOZ amplitude CFD (Figure 3B), suggesting the dominant cross‐frequency information flow from non‐SOZ HFA to SOZ LFA. Overall, the within‐frequency and cross‐frequency results indicated that the prevailing resting‐state information flow is always from non‐SOZ to SOZ.

### 2.2 Higher Excitation–Inhibition Imbalance in SOZ versus Non‐SOZ Revealed by 1/*f* Power Slope During the Resting State

**Figure 4:**
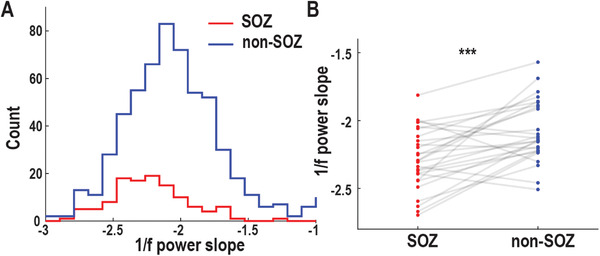
1/*f* power slope during the resting‐state. A) Distribution of 1/*f* power slope values shifts leftward (more negative) in SOZ (red) versus non‐SOZ (blue) electrodes. B) Individual‐patient comparison of averaged 1/*f* power slopes between SOZ (red) and non‐SOZ (blue), each patient represented by a pair of connected dots showing that the majority of patients (76.7%) had more negative slopes in SOZ compared to non‐SOZ. ***p < 0.001.

The differences in information flow between SOZ and non‐SOZ could be related to alternation in excitation/inhibition (E/I) balance. Based on computational modeling, it has been shown that the E:I ratio could be estimated from the 1/*f* power slope, in which the more negative power slope is associated with higher excitation–inhibition imbalance.^[^ ^24^ ^]^ Therefore, we investigated the 1/*f* power slope as an indicator of E:I imbalance. After computing the power spectrum between 1 and 250 Hz at each electrode, the 1/*f* power slope was derived with the FOOOF algorithm.^[^ ^25^ ^]^ Among 116 SOZ and 573 non‐SOZ electrodes, we found that power slopes of SOZ were significantly more negative than non‐SOZ electrodes (two‐sample *t*‐test, *p* < 10^−9^, **Figure** **4**A). On a single patient basis, 21 out of 27 patients had a more negative power slope in SOZ (Figure 4B). Taken together, SOZ had a more negative 1/*f* power slope in comparison to non‐SOZ during the resting state, probably reflecting the higher excitation–inhibition imbalance in SOZ.

### 2.3 Larger Resting‐State Information Flow Asymmetry Between SOZ and Non‐SOZ is Associated with Favorable Seizure Outcome

**Figure 5:**
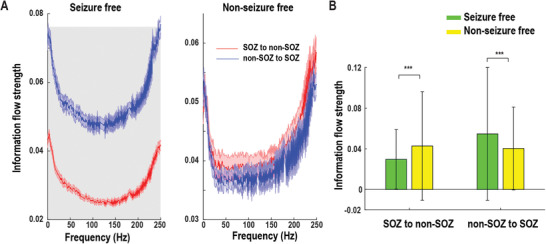
Association of resting‐state information flow with post‐seizure outcome. A) Within‐frequency information flow between SOZ and non‐SOZ according to seizure outcome. The shaded gray area indicates significant difference at the *p* = 0.05 level after FDR correction. Data are shown in mean and standard error. B) Averaged bidirectional within‐frequency information flow between SOZ and non‐SOZ over the broadband frequencies in (A). SOZ to non‐SOZ information flow strength was significantly weaker, but non‐SOZ to SOZ information flow strength was substantially stronger in seizure‐free than nonseizure free patients. Error bar represents standard deviation. ***p < 0.001.

Next, we investigated the association between the resting‐state connectivity and seizure outcome. Of these 27 patients, there were 19 patients with Engel I outcome (70.4%), 4 patients with Engel II outcome (14.8%), 3 patients with Engel III outcome (10.7%), and 1 patient with Engel IV outcome (3.7%). We classified Engel I outcome as seizure‐free and Engel II–IV outcome as the nonseizure free outcome. In the neural data, we found significant differences in within‐frequency bidirectional information flow between SOZ and non‐SOZ in the broadband frequency range in the seizure‐free patients. At the same time, there was no significant difference in nonseizure free patients (**Figure** **5**A). After averaging over the broadband frequencies, the differences in seizure outcome were driven by weaker SOZ to non‐SOZ information flow strength and stronger non‐SOZ to SOZ information flow strength in seizure‐free patients (Figure 5B). Note that we did not find such significant differences in the cross‐frequency information flow. Taken together, these suggested that larger resting‐state within‐frequency information flow asymmetry between SOZ and non‐SOZ was associated with favorable seizure outcome.

### 2.4 Individual Predictions of SOZ and Seizure Outcome with Random Forest Classifier

**Figure 6:**
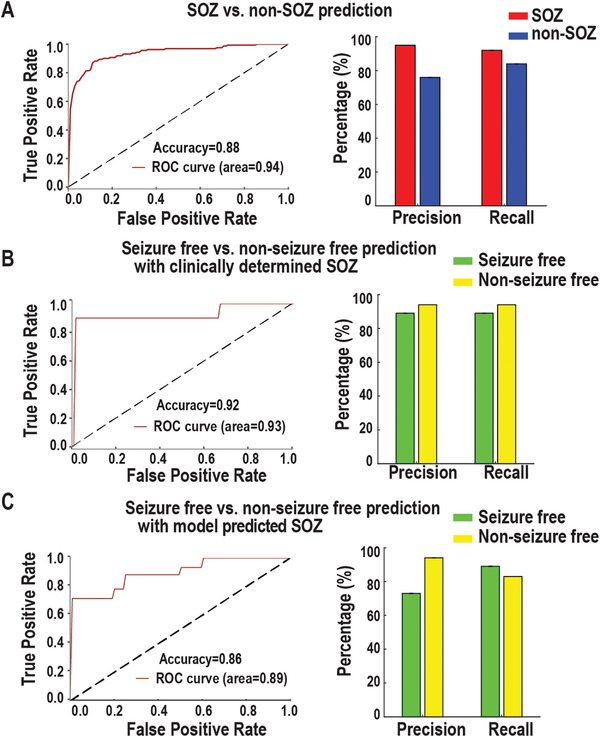
Performance of SOZ and seizure outcome predictions at the individual level. A) SOZ versus non‐SOZ prediction. Receiver‐operating characteristic (ROC) curves show the true‐positive and false‐positive rates in predicting SOZ versus non‐SOZ. The area under the curve (AUC) is 0.94. Precision = True Positive / (True Positive + False Positive); Recall = True Positive / (True Positive + False Negative). B) Similar to (A) but for prediction of seizure outcome with clinically determined SOZ, i.e., seizure‐free versus nonseizure free. C) Similar to (B) but with model predicted SOZ, where only 10 min resting state SEEG data were used.

Lastly, we utilized the random forest classifier to predict: 1) whether an individual electrode is likely to be SOZ; 2) whether the patient will be seizure‐free. Based on the statistical results above, within‐frequency interaction was significantly different in both information flow between SOZ and non‐SOZ comparison (Figure 2) and seizure‐free outcome versus nonseizure free outcome comparison (Figure 5). We only used the broadband within frequency information flow as feature inputs into the random forest classifier to increase interpretability. For SOZ prediction, the mean strength of within‐frequency inward information flow (1–250 Hz range in step of 1 Hz) at each electrode was computed as features. More specifically, each electrode has 250 features, which were obtained by averaging the within‐frequency inward information flow from all other electrodes in that patient. For outcome prediction, the strength of mean non‐SOZ to SOZ within‐frequency information flow (1‐250 Hz) over all non‐SOZ and SOZ paired electrodes for each patient was calculated as features. To evaluate random forest classifier's performance, we applied a fivefold cross‐validation approach. This approach randomly divided all samples into five subsets with each one consisting of the same proportion of each class label. Of the five subsets, four subsets (labels known) were used to train the model and the remaining one (unseen) was retained to test the model. This procedure was repeated five times until all subsets were used once as a testing test. As shown in **Figure** **6**A, the model demonstrated an accuracy of 0.88 and an AUC of 0.94 in predicting SOZ (Precision: 0.95; Recall: 0.76) versus non‐SOZ (Precision: 0.92; Recall: 0.84). For seizure outcome prediction, the model achieved an accuracy of 0.92 and an AUC of 0.93 when SOZ was identified by clinicians (Precision: [0.89 0.94]; Recall: [0.89 0.94] for seizure free and nonseizure free outcomes) (Figure 6B). If SOZ was estimated by our prediction model, the seizure outcome prediction accuracy was 0.86 with an AUC of 0.89 (Precision: [0.73 0.94]; Recall: [0.89 0.83] for seizure free and nonseizure free outcomes (Figure 6C). Overall, these findings suggest that the combination of random forest classifier and resting‐state connectivity may help identify SOZ and predict seizure outcome at the individual level with satisfactory accuracy.

Overall, our results suggest that the dominant information flow is always from non‐SOZ to SOZ at multiple frequencies in the interictal resting‐state period, which is probably due to the lower excitation–inhibition imbalance in non‐SOZ regions. Moreover, larger resting‐state information flow asymmetry between SOZ and non‐SOZ is associated with favorable seizure outcome. By incorporating both resting‐state connectivity and random forest classifier, it is possible to localize SOZ and predict seizure outcome at the individual level with satisfactory accuracy.

### 2.5 Multiple Oscillatory Push–Pull Antagonisms Underlying the Epilepsy Network

We hypothesized that there is competition in information flow between SOZ and non‐SOZ underlying epilepsy resting state (Figure S2, Supporting Information). There are two possibilities under our hypothesis: 1) SOZ tends to send more information to non‐SOZ; 2) non‐SOZ sends more information to SOZ. Our data show the dominant information flow from SOZ to non‐SOZ during the ictal period (Figure S1, Supporting Information) and the opposite pattern during the resting‐state (Figure 2). More specifically, SOZ received more information flow from non‐SOZ in a broadband frequency in the within‐frequency network (Figure 2), while the HFA of non‐SOZ sent information to the LFA of SOZ in the cross‐frequency network (Figure 3). Interestingly, a previous study has shown that the focal seizure propagation dynamic was constrained by push–pull antagonisms between SOZ and non‐SOZ.^[^ ^23^ ^]^ The modulation of non‐SOZ primarily determines whether the seizure propagates or not. During the resting state, non‐SOZ may send more directional information flow to SOZ to prevent seizure spread, probably reflecting the widespread network inhibition.^[^ ^26^ ^]^ Furthermore, more considerable asymmetry in resting‐state within‐frequency information flow between SOZ and non‐SOZ (weaker SOZ to non‐SOZ information flow and stronger non‐SOZ to SOZ information flow) was associated with favorable seizure outcome. These might suggest that the less capacity for SOZ to spread during the resting state, the more suppression from non‐SOZ to SOZ, the better seizure outcome.

### 2.6 Disrupted Excitation–Inhibition Balance in Epilepsy

Neural circuits rely on a dynamic E:I balance, and the balance of E:I interaction is critical for neuronal homeostasis and neural oscillation formation.^[^ ^27^ ^]^ Emerging evidence indicates that E:I balance has dynamically fluctuated with neural computation, task demands, and cognitive states.^[^ ^25^ ^]^ More dramatic changes and aberrant E:I patterns are implicated in neurological disorders such as epilepsy.^[^ ^28^ ^]^ The computation model developed by Gao et al. suggested that the E:I ratio can be quantified from the power spectrum, with a flatter 1/*f* power slope (less negative value) indicating a lower E:I imbalance.^[^ ^24^ ^]^ This was supported by the evidence that 1/*f* power slope tracked the propofol‐induced global inhibition, in which significant slope decrease was observed during anesthesia when compared to awake. In our data, 1/*f* power slope changes significantly between the resting‐state and ictal state (Figure S3, Supporting Information), indicting different levels of excitation–inhibition imbalance. Moreover, a more negative power slope in SOZ was found when comparing to non‐SOZ during the resting state (Figure 4), probably reflecting high excitation–inhibition imbalance in SOZ but low excitation–inhibition imbalance in non‐SOZ. The disrupted excitation–inhibition balance might be linked to our findings in information flow. The non‐SOZ with low excitation–inhibition imbalance could be the source of information sender to SOZ with high excitation–inhibition imbalance, explaining why the dominant information is from non‐SOZ to SOZ. However, it is not satisfactory to predict SOZ at the individual electrode level using 1/*f* power slope as features (Figure S4, Supporting Information), in which the model demonstrated an accuracy of 0.56 and an AUC of 0.58 in predicting SOZ (Precision: 0.40, Recall: 0.79) versus non‐SOZ (Precision: 0.20, Recall: 0.66).

### 2.7 Long‐Range Communication Disruption of High‐Frequency Activity in the Epilepsy Network

**Figure 7:**
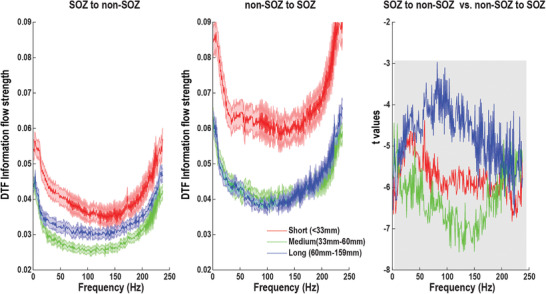
Within‐frequency directional information flow as a function of distance during the resting state. Left panel: Mean SOZ to non‐SOZ DTF information flow of all electrode pairs in distance‐range quartiles. Middle panel: Mean non‐SOZ to SOZ DTF information flow of all electrode pairs in distance‐range quartiles. Right panel: statistical difference between SOZ to non‐SOZ DTF information flow and non‐SOZ to SOZ DTF information flow in distance‐range quartiles. Significant area at the *p* = 0.05 level after FDR correction is marked in shadow. Data are shown in mean and standard error.

Synchronization between neuronal populations is critical for information transfer between brain areas.^[^ ^29^ ^]^ Theoretical and experimental evidence has shown that synchronization between neuronal oscillations depend on the axonal conduction delays, so LFA are generally more stably synchronized over long distance than HFA.^[^ ^30^ ^]^ Surprisingly, our data challenged this classical view and demonstrated HFA exhibiting long‐range communication both within SOZ, within non‐SOZ (Figure S5, Supporting Information), and between SOZ and non‐SOZ (**Figure** **7**). We estimated the information flow using DTF between all SEEG electrode pairs from 1 to 250 Hz and divided them into three groups based on distances. If the distance between paired electrodes is shorter than 33 mm, it is termed as short‐range connection while it is long‐range connection if the distance is longer than 60 mm. DTF information flows were first averaged over all patients in three quartiles of interelectrode distances. The mean DTF information flow increased from 1 to 6 Hz in all distance quartiles and then decayed to 80 Hz. However, throughout the 80–250 Hz HFA, inter‐electrode DTF information flow started to increase again and exhibited a peak at around 240 Hz. Note that the long‐range communication coordinated by HFA was shown in a recent study in healthy brain regions.^[^ ^31^ ^]^ Here, we extended the previous findings and provided the first evidence of long‐range communication disruption of HFA between SOZ and non‐SOZ in the pathological epilepsy network. The long‐range neuronal communication of HFA could arise in large‐scale network because the joint roles of local synchronization and high collective firing rates enable local pyramidal cell populations with largely increased efficiency in regulating their post‐synaptic targets in distant regions.^[^ ^32^ ^]^ This could be experimentally observable as inter‐areal HFA phase coupling and would constitute a direct indication of spiking based long‐range neuronal communication per se.

### 2.8 Influences of MRI Findings and Outcome in the Model

The performance of the prediction model could be influenced by the MRI findings and outcome of the epilepsy patients. In our cohort, it had 15 nonlesional patients and 12 lesional patients (9 patients with focal lesions and 3 patients with diffuse lesions). Our SOZ prediction model works best in the patients without MRI visible lesions (Precision: 0.94, Recall: 0.89) followed by the patients with focal lesions (Precision: 0.81, Recall: 0.83) and then the patients with diffuse lesions (Precision: 0.69, Recall: 0.83) (Figure S6A, Supporting Information). When statistically comparing the 1/*f* power slope between the lesional and nonlesional groups (Figure S6B, Supporting Information), they are not significantly different in both SOZ (lesional group: −2.23 ± 0.33, nonlesional group: −2.30 ± 0.30; *t*(114) = 1.16, *p* = 0.24) and non‐SOZ (lesional group: −2.08 ± 0.44, nonlesional group: −2.07 ± 0.38; *t*(571) = −0.48, *p* = 0.63). When patients were divided by outcome, the overlap between our model estimated SOZ and clinically determined SOZ was significantly lower in the nonseizure free group (Mean = 0.78, SD = 0.22) than the seizure free group (Mean = 0.85, SD = 0.19) (*t*(25) = ‐2.19, *p* = 0.038, Figure S7A, Supporting Information), suggesting the epileptogenic zone in the nonseizure free group may not be properly targeted during surgery or intervention. Besides, the performance of concordance in detecting the SOZ at the individual patient level is shown in Figure S7B (Supporting Information), in which the sensitivity and precision were higher in the seizure free group (Sensitivity: Mean = 0.85, Range = [0.5 1],Precision: Mean = 0.88, Range = [0.62 1]) compared to the nonseizure free group (Sensitivity: Mean = 0.80, Range = [0.33 1]; Precision: Mean = 0.75, Range = [0.57 1]). However, the specificity was lower in the seizure free group (Specificity: Mean = 0.91, Range = [0.77 1]) when comparing to the nonseizure group (Sensitivity: Mean = 0.93, Range = [0.84 1]).

### 2.9 Stability of the Results During Long‐Term Implantation

The short duration interictal resting state data represent only snapshots of the long‐term implantation period, while studies have shown temporal variations of the epileptiform activity during longer periods of time.^[^ ^33^ ^]^ Thus, the stability of the results for several intervals on different days is under question. For example, antiepileptic drugs (AEDs) are gradually withdrawn to facilitate seizure occurrence during the SEEG implantation, do the results change with respect to AED withdrawal strategy? To address these questions, we performed several control analyses. First, we showed that our findings are stable and robust for all patients under different durations with 5, 10, 15, and 30 min (Figure S8, Supporting Information). Then, we selected 5 patients and extracted 10 min resting‐state data on 3 different time periods of a day (Early Morning/Afternoon/ Evening) and 3 different days (Day2/Day3/Day4) during the implantation period. After calculating the 1/*f* power slope and the within‐frequency information flows, the grand average results of these patients are shown in Figures S9 and S10 (Supporting Information). Although fluctuations do exist, the results were quite stable over different intervals on different days.

### 2.10 Contributions of Resting‐State Data in Clinical Decision Making

Ultimately, this study aims to improve seizure outcome of epilepsy patients, which largely relies on the precise delineation of epileptic networks. Here, we demonstrated that our approach could predict both SOZ and seizure outcome with about 90% accuracy in a large cohort of 27 drug‐resistant focal epilepsy patients using a few minutes of interictal intracranial EEG resting‐state data. In classical neuroimaging studies, resting state is the period when participant is awake and not performing an explicit mental or physical task. In our study, resting state refers to interictal period without visible pathological activity. We selected epochs recorded between 7:00AM and 12:30 PM, during which patients were most likely in eyes‐open and awake condition. The interpretations of ictal data have limitations, mainly imposed by accelerated meditation changes during the intracranial study and electrode coverage leading to sampling biases that may affect localization accuracy.^[^ ^9^, ^34^ ^]^ Moreover, it is challenging to capture all types of seizures during hospitalization.^[^ ^35^ ^]^ For example, one study showed that approximately one‐third of bilateral temporal lobe epilepsy patients required more than four weeks of recordings to capture bilateral independent seizures.^[^ ^36^ ^]^ In addition, seizure clusters may provide discordant data that may misdirect interpretation and surgical treatment.^[^ ^37^ ^]^ However, it should be pointed out that we explored the possibility to localize SOZ and predict seizure outcome using short duration interictal recordings while the golden standard of localization of epileptogenic brain regions in current clinical practice still depends on capturing multiple seizures during the intracranial monitoring process. We believe our method could aid clinicians estimate the location of SOZ and assess the likelihood of benefit from surgery in an automatic and objective way, which has the potential to improve the current practice. For example, if the preoperative prediction of seizure outcome for a specific patient from our model is nonseizure free, it would suggest nonusual resection or other treatment option.

The underlying pathology of seizure generation most likely involves both abnormal brain structures and aberrant connectivity among these regions, leading to large‐scale network disruptions.^[^ ^19b^ ^]^ The aberrant network connectivity could be studied under resting‐state, and many resting‐state intracranial EEG studies have shown overwhelmingly enhanced connectivity involving EZ and surrounding regions.^[^ ^38^ ^]^ Verhoeven et al. used directed functional connectivity patterns estimated during EEG periods without visible pathological activity to automatically diagnose and lateralized temporal lobe epilepsy (TLE). The diagnosis and lateralization classifiers achieved a high accuracy (90.7% and 90.0%, respectively) and the most important features for diagnosis were the outflows from left and right medial temporal lobe, and for lateralization the right anterior cingulate cortex.^[^ ^39^ ^]^ It should be pointed out that our study aimed to predict SOZ while Verhoeven et al. tried to distinguish TLE versus healthy controls and left versus right TLE. More recently, two SEEG resting‐state studies suggested the possibility to predict SOZ/EZ at the individual electrode level.^[^ ^10a,b^ ^]^ Goodale et al. computed 8–12 Hz alpha‐band imaginary coherence across all electrodes using 2 min resting‐state SEEG data in a cohort of 15 adult focal epilepsy patients.^[^ ^10a^ ^]^ Six functional connectivity measures were incorporated in the logistic regression model to predict epileptogenicity of individual regions, and their model showed an AUC of 0.78 and an accuracy of 80.4%. Narasimhan et al. investigated 25 focal epilepsy patients with 2 min of resting‐state, artifact‐free SEEG data, and calculated three nondirected connectivity measures and four directed measures in the alpha band.^[^ ^10b^ ^]^ Logistic regression was further applied to generate a predictive model of ictogenicity with an AUC of 0.88 and an accuracy of 84.3%. In our work, we used 10 min resting‐state SEEG data to predict both SOZ and seizure outcome in a cohort of 27 epilepsy patients. We investigated both within‐frequency and cross‐frequency directional connectivity network during a wide frequency range from 1 to 250 Hz. To tackle the problem of severely imbalanced data between SOZ and non‐SOZ, a balanced random forest model was introduced by optimizing the cost function and the sampling technique. Our results, obtained from a relatively large patient population using a network connectivity approach, demonstrated enhanced performance of localizing SOZ with an AUC of 0.94 and an accuracy of 0.88. Besides, we examined the E:I ratio by computing the 1/*f* power slope and provided deeper mechanistic insights between the E:I alteration and aberrant connectivity in the resting‐state epilepsy network. Furthermore, by utilizing directional connectivity network information, we made important advancement to predict seizure outcome with satisfactory accuracy. Prospective validation of our findings would pave the way to reducing traditional prolonged seizure recordings, leading to shorter hospitalizations and improved patient care.

### 2.11 Study Limitations

One limitation of this study is that we used only the clinical SOZ to approximate the EZ concept. This is a limitation because the SOZ can be a subset of the EZ, but the fact that only a fraction of our patients achieved seizure‐freedom (i.e., Engel I). Quantitative methods such as fingerprint have been developed to objectively delineate EZ.^[^ ^17^, ^40^ ^]^ The EZ fingerprint as a time‐frequency pattern that is defined by a combination of preictal spike(s), fast oscillatory activity, and concurrent suppression of lower frequencies. Not surprisingly, the fingerprint estimated EZ and clinician determined SOZ is largely overlapped (Overlap percentage: 71.29% ± 10.22%). When using fingerprint estimated EZ to predict seizure outcome (Figure S11, Supporting Information), the model achieved an accuracy of 0.77 and an AUC of 0.78 (Precision: [0.73 0.82]; Recall: [0.86 0.67] for seizure free and nonseizure free outcomes), which was not as good as clinician determined SOZ and our model predicted SOZ. Besides, the seizure typically originates in the SOZ and propagates to the propagation zone (PZ), which is often presented in the SEEG recordings. Here, we used SOZ to represent EZ and attributed the PZ to non‐SOZ in our model to make the framework more concise and easier to interpret. When comparing the E:I ratio inferred by 1/*f* power slope between different zones (Figure S12, Supporting Information), PZ has a more negative 1/*f* power slope than other zones (*t*(573) = –3.68, *p* < 0.001), but less negative 1/*f* power slope than SOZ (*t*(205) = –2.12, *p* < 0.05). Another limitation stems from the short 10‐min artifact and spike‐free interictal data selection for our analysis. The selected recordings were visually examined for the presence of epileptiform activity or significant spiking activity, while it was still possible that some spiking activity may exist. Azeem et al. showed that interictal spike networks predict surgical outcome in patients with drug‐resistant focal epilepsy.^[^ ^41^ ^]^ To assess the influence of interictal spikes on our results, we selected 10 min continuous interictal recordings (not excluding spikes) for all patients and re‐performed the SOZ and seizure outcome prediction analysis. As shown in Figure S13A (Supporting Information), the SOZ model demonstrated an accuracy of 0.86 and an AUC of 0.81 in predicting SOZ (Precision: 0.85, Recall: 0.89) vs non‐SOZ (Precision: 0.81, Recall: 0.87). For seizure outcome prediction (Figure S13B, Supporting Information), the model achieved an accuracy of 0.89 and an AUC of 0.90 for seizure free (Precision: 0.92, Recall: 0.86) and nonseizure free (Precision: 0.90, Recall: 0.88) outcomes. Therefore, the performances of SOZ and outcome prediction with or without spikes are comparable and quite similar, suggesting that spikes have little influence on our results. Besides, we did not control patients’ state specifically, while behavior states such as eyes open/eyes closed and sleep/awake conditions could influence our findings. However, the results from data during morning, afternoon and evening remained unchanged (Figure S10, Supporting Information), indicating that our findings may be independent of behavior states. Moreover, we only used within‐frequency information flow as features to make an individual prediction of seizure outcome for simplicity and more straightforward interpretation. We argued that network dynamics may be inherited properties that would impact outcome. Besides, we did not take the resection location and extent into account in our model because we would like to make prediction before the surgery actually performed. It is also important to note that a few clinical variables such as absence of generalized tonic‐clonic seizures and presence of hippocampal atrophy were significantly associated with seizure remission.^[^ ^42^ ^]^ Future studies will be needed to develop multivariate outcome prediction models by taking clinical variables into account. Lastly, the majority of patients (19 out of 27) are temporal lobe epilepsy patients. It should be cautious to generalize our findings to all candidates for epilepsy surgery.

## 3 Conclusion

We have investigated the aberrant information flow and the disrupted excitation–inhibition balance in epilepsy resting state during interictal period. We found that the lower excitation–inhibition imbalance in non‐SOZ versus SOZ regions during the resting state could be linked to the dominant information flows from non‐SOZ to SOZ at multiple frequencies, probably reflecting insufficient excitability to initiate seizure and widespread network inhibition to prevent seizure initiation. Moreover, stronger resting‐state information flow from non‐SOZ to SOZ was found in seizure free patients compared to nonseizure free patients. In combination with the balanced random forest machine learning model and resting‐state connectivity, localization of SOZ and seizure outcome prediction without long‐term recordings may supplement traditional interpretation of SEEG and help identify epilepsy treatment targets, thus improving patient care and treatment outcome.

## 4 Experimental Section

### Patients

The study included 27 drug‐resistant focal epilepsy patients who underwent complete presurgical evaluation, including SEEG at the University of Pittsburgh Medical Center between 2014 and 2019. All patients enrolled during the period with SEEG recordings were considered and patients with clear recordings of SOZ were included. Demographic and clinical information of patients is summarized in Table S1 (Supporting Information). Treatments such as surgical resection (19 patients) and ablation (8 patients) were conducted during the medical care. Besides, postoperative seizure outcome was evaluated at the last follow‐up (>1 year) using the Engle classification scale.^[^ ^43^ ^]^ This study was approved by local institutional review boards at the University of Pittsburgh and Carnegie Mellon University (STUDY2018_00000097). Written informed consent was obtained from all patients.

### SEEG Data Collection

The SEEG data were recorded using the Xltek acquisition system (Natus Medical Inc, Pleasanton, CA) with a 2 kHz or 1 kHz (3 patients) sampling rate. Ten‐minute epochs were randomly selected from the long‐term SEEG recordings during interictal periods in which the patient was at rest. All selected epochs were recorded between 7:00AM and 12:30PM, and at least 2 h away from an ictal event. Each selected recording segment was about 3 min long and visually examined for the presence of epileptiform activity or significant spiking activity. Following this procedure, these segments were concatenated and merged into various length. Raw data were notch filtered at 60 Hz and re‐referenced using bipolar montage. Electrode pairs residing in white matter were excluded from further analysis.

SOZ was marked by board‐certificated epileptologists using established clinical interpretation. The SOZ determination occurred during the intracranial recording session and was completed before any data analysis in this study. The seizure onset was indicated by a variety of stereotypical electrographic features, which include, but were not limited to, the onset of fast rhythmic activity, an isolated spike or spike and wave complex followed by rhythmic activity, or an electrodecremental response.^[^ ^44^ ^]^ Besides, one typical focal seizure for each patient was extracted for the control analysis.

### Directional Connectivity Estimation

The within‐frequency and cross‐frequency directional information flow were estimated by means of the directed transfer function (DTF)^[^ ^45^ ^]^ and cross‐frequency directionality (CFD)^[^ ^22^, ^23^ ^]^ respectively. Based on the framework of multivariate autoregressive (MVAR) models, DTF provides a spectral measure for directed information flow in the spectral domain in the multivariate system.^[^ ^21^, ^45^ ^]^ It has been demonstrated that DTF was useful in objectively determining underlying pathological connections such as epilepsy.^[^ ^46^ ^]^ The DTF analysis was conducted using the open‐source toolbox Fieldtrip,^[^ ^47^ ^]^ which is freely available at https://www.fieldtriptoolbox.org/.

Since DTF is only able to estimate the directional information flow at a single frequency, CFD is further utilized to quantify the directional interactions between different frequencies.^[^ ^22^ ^]^ CFD has been applied in different electrophysiological modalities, including magnetoencephalography,^[^ ^48^ ^]^ electroencephalography,^[^ ^49^ ^]^ and electrocorticography,^[^ ^23^, ^50^ ^]^ often revealing new insights into multilayer network interactions. The core basis of CFD is the phase‐slope index (PSI), assuming that constant lag in the time lag could be represented by linearly increasing or decreasing phase differences in the considered frequency range.^[^ ^51^ ^]^ By computing the PSI between the phase of low‐frequency activity (LFA) (< 30 Hz) and the amplitude of high‐frequency activity (HFA) (> 30 Hz), the positive CFD indicates information flow from LFA to HFA and vice versa for the negative CFD.

### 1/*f* Power Slope Estimation

The power‐law exponent (slope) of the power spectrum (1/*f*) has been suggested to estimate synaptic excitation (*E*) – inhibition (*I*) ratios and changes dynamically under different states.^[^ ^24^ ^]^ Here, the 1/*f* power slope with FOOOF package (https://github.com/fooof‐tools/fooof) is estimated. To obtain power spectrum, data were epoched into 1 s segment without overlapping, and the time‐frequency decomposition was estimated by a Fast Fourier Transformation in combination with a Hanning taper from 1 to 250 Hz in 1 Hz step. Then, the power spectrum of each epoch was computed and subsequently averaged over all epochs. After the power spectrum calculation, the FOOOF algorithm operates on power spectrum densities in semilog‐power space, which are linearly spaced frequencies and log‐spaced power values.^[^ ^25^ ^]^ Essentially, the 1/*f* power slope is fit as a function across the selected range of the spectrum, and each oscillatory peak is modeled with a Gaussian function individually.

### Random Forest Classification

To predict at the individual electrode level (e.g., SOZ vs non‐SOZ) and patient‐level (e.g., seizure‐free vs nonseizure free outcome), the random forest machine learning technique was utilized. Random forest is an ensemble machine learning method that induces each constituent decision tree from bootstrap samples of the training data.^[^ ^52^ ^]^ The prediction is made by aggregating all decision trees' predictions. For SOZ individual prediction, the majority of electrodes are non‐SOZ. Thus, these two classes (SOZ and non‐SOZ) are severely imbalanced. Dealing with highly imbalanced data, a sample may contain few or even none of the minority class, resulting in a tree with a poor predicting performance for the minority class. To tackle the problem of severely imbalanced data, a balanced random forest was introduced by adopting two strategies: 1) minimizing the overall cost by assigning a high cost to the misclassification of minority class; 2) either oversampling the minority class or downsampling the majority class or both.^[^ ^53^ ^]^ Here, a synthetic minority oversampling technique (SMOTE) was applied, a combination of oversampling the minority class and undersampling the majority class.^[^ ^54^ ^]^ The balanced random forest is implemented in open‐source python toolbox imbalanced‐learn (https://github.com/scikit‐learn‐contrib/imbalanced‐learn) and adapted in the study.^[^ ^55^ ^]^ To evaluate the model's performance, the five‐fold cross‐validation approach was applied, further generated the receiver operator characteristic (ROC) curve, and computed the area under the curve (AUC). The metrics of precision, recall, and overall accuracy were also assessed.

## Conflict of Interest

H.J. and B.H. are inventors on an U.S. provisional patent application submitted by Carnegie Mellon University that covers some analysis techniques used in this work. All other authors declare that they have no competing interests.

## Author Contributions

H.J. and B.H. conceived the idea. H.J. and B.H. designed the study and wrote the manuscript draft. H.J. analyzed the data. M.R., A.B., V.K., A.U. collected the data. S.Y., and V.K. prepared and preprocessed the data. M.R., A.B., V.K., and S.Y. revised the manuscript. All the authors discussed the results and contributed to the manuscript. B.H. supervised the research.

## Supporting information

Supporting Information

## Data Availability

The data that support the findings of this study are available from the corresponding author upon reasonable request.
