# Supplementary material for: Interictal SEEG Resting‐State Connectivity Localizes the Seizure Onset Zone and Predicts Seizure Outcome
Source: Adv Sci (Weinh). 2022 May 12;9(18):2200887. doi: 10.1002/advs.202200887 (PMC9218648; doi:10.1002/advs.202200887)
Supplement: Supplementary file 1 — Supporting Information [file ADVS-9-2200887-s001.pdf]

ADVANCED SCIENCE
Open Access

Supporting Information

for *Adv. Sci.*, DOI 10.1002/advs.202200887

Interictal SEEG Resting-State Connectivity Localizes the Seizure Onset Zone and Predicts Seizure Outcome

*Haiteng Jiang, Vasileios Kokkinos, Shuai Ye, Alexandra Urban, Anto Bagić, Mark Richardson and Bin He**

Supplementary Materials

for

Interictal SEEG resting-state connectivity localizes the seizure onset zone and predicts seizure outcome

Haiteng Jiang, PhD[1,2,3], Vasileios Kokkinos, PhD[4,5], Shuai Ye, MS[1], Alexandra Urban, MD[4], Anto Bagić, MD, PhD[4], Mark Richardson, MD[4,5], and Bin He, PhD[1,6]*

1. Department of Biomedical Engineering, Carnegie Mellon University, Pittsburgh, PA, USA
2. Department of Neurobiology, Affiliated Mental Health Center & Hangzhou Seventh People's Hospital, Zhejiang University School of Medicine, Hangzhou, China
3. NHC and CAMS Key Laboratory of Medical Neurobiology, MOE Frontier Science Center for Brain Science and Brain-machine Integration, School of Brain Science and Brain Medicine, Zhejiang University, Hangzhou, China
4. University of Pittsburgh Comprehensive Epilepsy Center, Department of Neurology, University of Pittsburgh School of Medicine, Pittsburgh, PA, USA
5. Massachusetts General Hospital, Boston, MA, USA
6. Neuroscience Institute, Carnegie Mellon University, Pittsburgh, PA, USA

* Correspondence:
Bin He, PhD
Department of Biomedical Engineering
Carnegie Mellon University
5000 Forbes Avenue, Pittsburgh, PA 15213
e-mail: bhe1@andrew.cmu.edu

Supplementary Methods

Directed transfer function connectivity estimation

The direct transfer function (DTF) was applied to estimate the within-frequency directed information flow, or directed functional connectivity [1]. For a multivariate time series $Y(t)$, it can be described by multivariate adaptive auto-regression (MVAR) as follows:

$$Y(t) = \sum_{k=1}^{p} A(k)Y(t-k) + E(t) \quad \text{(S-1)}$$

where $A(k)$ is the coefficient matrix, $E(t)$ is multivariate independent white noise, and p is the model order. The transfer function was derived by taking the Fourier transform of Eq. (S-1) and then inverting the coefficient matrix, which can be described as follows:

$$A(f)Y(f) = E(f)$$

$$Y(f) = A^{-1}(f)E(f) = H(f)E(f) \quad \text{(S-2)}$$

where $A(f) = \sum_{k=0}^{p} A_k e^{-j2\pi f \Delta t k}$ and $A_{k=0} = I$. Here, A is the coefficient matrix obtained from the MVAR model, and $H(f)$ is the transfer matrix, which is the inverse of the coefficient matrix and contains frequency-specific directional interaction information. The DTF value $\gamma_{ij}(f)$ represents the information flow from node i to node j, and is typically normalized by dividing each element by the sum of each row

$$\gamma_{ij}^{2}(f) = \frac{|H_{ij}(f)|^2}{\sum_{m=1}^{n} |H_{im}(f)|^2} \quad \text{(S-3)}$$

with the normalization condition $\sum_{k=1}^{n} \gamma_{ij}^{2}(f) = 1$, where n is the number of investigated frequency bins.

Cross-frequency directionality analysis

Cross-frequency directionality (CFD) can be used to evaluate directional interactions between different frequencies by computing the phase-slope index (PSI) between the phase of low frequency activity (LFA) and the amplitude envelope of high frequency activity (HFA) [2].

Let $\boldsymbol{x}^s$ denote the raw signal at segment s and $\boldsymbol{y}^{v,s}$ denote the amplitude envelope at high frequency v. We define $\boldsymbol{X}^s$ and $\boldsymbol{Y}^{v,s}$ as the Fourier transforms of $\boldsymbol{x}^s$ and $\boldsymbol{y}^{v,s}$ respectively, and $\boldsymbol{\xi}^{v,s} = \boldsymbol{X}^s\overline{\boldsymbol{Y}^{v,s}}$ is the cross-spectrum between $\boldsymbol{X}^s$ and $\boldsymbol{Y}^{v,s}$, where '$-$' defines the complex conjugate. Let the complex coherency be defined as

$$C(v,f) = \frac{\sum_{s=1}^{S} \boldsymbol{X}^s(f)\left(\boldsymbol{Y}^{v,s}(f)\right)^*}{\sqrt{\sum_{s=1}^{S} |\boldsymbol{X}^s(f)|^2 \sum_{s=1}^{S} |\boldsymbol{Y}^{v,s}(f)|^2}} \quad \text{(S-4)}$$

The CFD between signal $\boldsymbol{x}$ and the amplitude envelope of the signal $\boldsymbol{y}^v$ at frequency tile (v,f) is defined as:

$$\Psi(v,f) = \operatorname{Im}\left(\sum_{g\in\mathcal{F}} C(v,g)^* C(v,g+\Delta f)\right) \quad \text{(S-5)}$$

with $\mathcal{F} = \left\{f - \frac{\beta}{2}, f - \frac{\beta}{2} + \Delta f, \dots, f + \frac{\beta}{2} - \Delta f\right\}$ where Δf is the frequency resolution, β is the bandwidth used to calculate the phase slope and Im denotes the imaginary part. The intuition of CFD algorithm is illustrated in Figure S14.

For the CFD calculations, the high-frequency amplitude envelope was extracted using a sliding time window approach by applying a discrete Fourier transform to successive data segments after applying a Hanning taper (5 cycles long concerning the frequency of interest). This was done from 20 to 250 Hz in steps of 5 Hz. The bandwidth β for estimating the PSI was set to 2 Hz surrounding the central phase frequencies of 2 to 20 Hz in 1 Hz steps to calculate CFD.

1/f power slope estimation

The power slope was estimated using a parameterization method in the FOOOF Python module (https://github.com/fooof-tools/fooof). This algorithm models the power spectral density (PSD) as a combination of periodic and aperiodic components [3], which can be described as follows:

$$PSD = L + \sum_{n=0}^{N} G_n \quad \text{(S-6)}$$

where L is the 1/f aperiodic component, G_n is a Gaussian to model each periodic component, and N is the total number of periodic components. The aperiodic component L is further modeled using a Lorentzian function as follows:

$$L = b - \log(k + F^{\lambda}) \quad \text{(S-7)}$$

where b is the offset, F is the vector of input frequencies, χ is the exponent, and k is the 'knee' parameter, controlling for the bend in the aperiodic component [4]. When there is no knee, there is a direct relationship between the slope, a of the line in log-log spacing, and the exponent χ, which χ=-a. To account for the effects of outliers on linear fitting and have an unbiased estimation, the FOOOF algorithm introduces a two-step strategy by finding an initial fit of the aperiodic component and identifying only the data points along the frequency axis that are most likely not to be part of periodic components. A second fit of the original PSD is then performed only on these frequency points, giving a more robust estimation of the aperiodic component.

References

[1] a) M. Kaminski, M. Ding, W. A. Truccolo, S. L. Bressler, *Biol. Cybern.* **2001**, *85* (2), 145, https://doi.org/10.1007/s004220000235; b) B. He, L. Astolfi, P. A. Valdes-Sosa, D. Marinazzo, S. Palva, C. G. Benar, C. M. Michel, T. Koenig, *IEEE Trans. Biomed. Eng.* **2019**, https://doi.org/10.1109/TBME.2019.2913928.

[2] a) H. Jiang, A. Bahramisharif, M. A. van Gerven, O. Jensen, *NeuroImage* **2015**, *118*, 359, https://doi.org/10.1016/j.neuroimage.2015.05.044; b) G. Nolte, A. Ziehe, V. V. Nikulin, A. Schlogl, N. Kramer, T. Brismar, K. R. Muller, *Phys. Rev. Lett.* **2008**, *100* (23), 234101, https://doi.org/10.1103/PhysRevLett.100.234101.

[3] T. Donoghue, M. Haller, E. J. Peterson, P. Varma, P. Sebastian, R. Gao, T. Noto, A. H. Lara, J. D. Wallis, R. T. Knight, A. Shestyuk, B. Voytek, *Nat. Neurosci.* **2020**, *23* (12), 1655, https://doi.org/10.1038/s41593-020-00744-x.

[4] a) R. Gao, R. L. van den Brink, T. Pfeffer, B. Voytek, *Elife* **2020**, *9*, https://doi.org/10.7554/eLife.61277; b) K. J. Miller, L. B. Sorensen, J. G. Ojemann, M. den Nijs, *PLoS Comput. Biol.* **2009**, *5* (12), e1000609, https://doi.org/10.1371/journal.pcbi.1000609.

Supplementary Table

Table S1 Demographic and clinical information of patients

Patient ID	Gender (M/F)	Onset (year)	Duration (year)	Epilepsy Type	Recording Electrodes	MRI Findings	Treatment	Outcome (Engel)
EP1055	M	20	15	Unilateral mesial temporal	11 electrodes, 87 contacts	Non-lesional	Resection	I C
EP1062	F	48	2	Unilateral mesial temporal	8 electrodes, 40 contacts	Non-lesional	Resection	I B
EP1082	M	20	37.8	Focal neocortical	11 electrodes, 112 contacts	No acute intracranial abnormality. Stable left frontal post-surgical encephalomalacia.	Resection	I A
EP1083	M	38	3.8	Unilateral mesial temporal	13 electrodes, 108 contacts	Left mesial temporal sclerosis	Resection	I A
EP1092	M	27	3.5	Unilateral mesial temporal	14 electrodes, 118 contacts	Right lateral orbitofrontal abnormality (heterotopia vs ischemic vs traumatic injury)	Resection	IV B
EP1095	F	7	35.2	Unilateral mesial temporal	8 electrodes, 64 contacts	Right mesial temporal sclerosis	Resection	I B
EP1098	F	2	20.3	Unilateral mesial temporal	15 electrodes, 140 contacts	Right temporo-parietal porencephalic cyst	Resection	III A
EP1103	F	34	12.5	Bilateral mesial temporal	16 electrodes, 160 contacts	Non-lesional	Resection	I A
EP1109	M	35	8.6	Unilateral mesial temporal	7 electrodes, 62 contacts	Right mesial temporal sclerosis	Resection	II B
EP1111	F	28	27.7	Unilateral mesial temporal	17 electrodes, 126 contacts	Bilateral mesial temporal sclerosis	Resection	III A
EP1115	F	5	38.8	Bilateral mesial temporal	9 electrodes, 72 contacts	Non-lesional	Resection	I A
EP1136	F	1.1	50	Bilateral mesial temporal	15 electrodes, 104 contacts	Non-lesional	Ablation	I A
EP1137	F	21	2.9	Unilateral mesial temporal	11 electrodes, 98 contacts	Tuberus sclerosis post left anterior temporal lobectomy.	Ablation	II A
EP1163	M	13	22.9	Bilateral mesial temporal	18 electrodes, 150 contacts	Right mesial temporal sclerosis	Resection	I A
EP1179	M	7	20.3	Unilateral mesial temporal	15 electrodes, 152 contacts	Non-lesional	Ablation	II B
EP1116	M	10	35.8	Focal neocortical	16 electrodes, 104 contacts	Non-lesional	Resection	I A
EP1120	F	22	8	Unilateral mesial temporal	13 electrodes, 106 contacts	Non-lesional	Resection	I A
EP1130	F	31	5.9	Bilateral mesial temporal	18 electrodes, 136 contacts	Left mesial temporal sclerosis and left parieto-occipital encephalomalacia post resection.	Ablation	I B
EP1131	F	8	52.5	Unilateral	12 electrodes,	Non-lesional	Resection	I A

				mesial temporal	86 contacts			
EP1132	M	1.2	20.4	Bilateral mesial temporal	8 electrodes, 60 contacts	Non-lesional	Resection	III A
EP1148	M	15	7.3	Focal neocortical	9 electrodes, 90 contacts	Bilateral occipital heterotopia.	Resection	I A
EP1149	F	32	6.5	Bilateral mesial temporal	14 electrodes, 122 contacts	Non-lesional	Ablation	I A
EP1164	M	16	3.9	Unilateral mesial temporal	17 electrodes, 208 contacts	Non-lesional	Ablation	II A
EP1170	M	20	8.1	Unilateral mesial temporal	11 electrodes, 136 contacts	Non-lesional	Ablation	I A
EP1126	F	16	20.4	Unilateral mesial temporal	10 electrodes, 56 contacts	Non-lesional	Resection	I C
EP1167	M	2	47.1	Focal neocortical	13 electrodes, 144 contacts	Non-lesional	Ablation	I A
EP1085	M	30	26.9	Unilateral mesial temporal	9 electrodes, 76 contacts	Left mesial temporal sclerosis	Resection	I A

Supplementary Figures

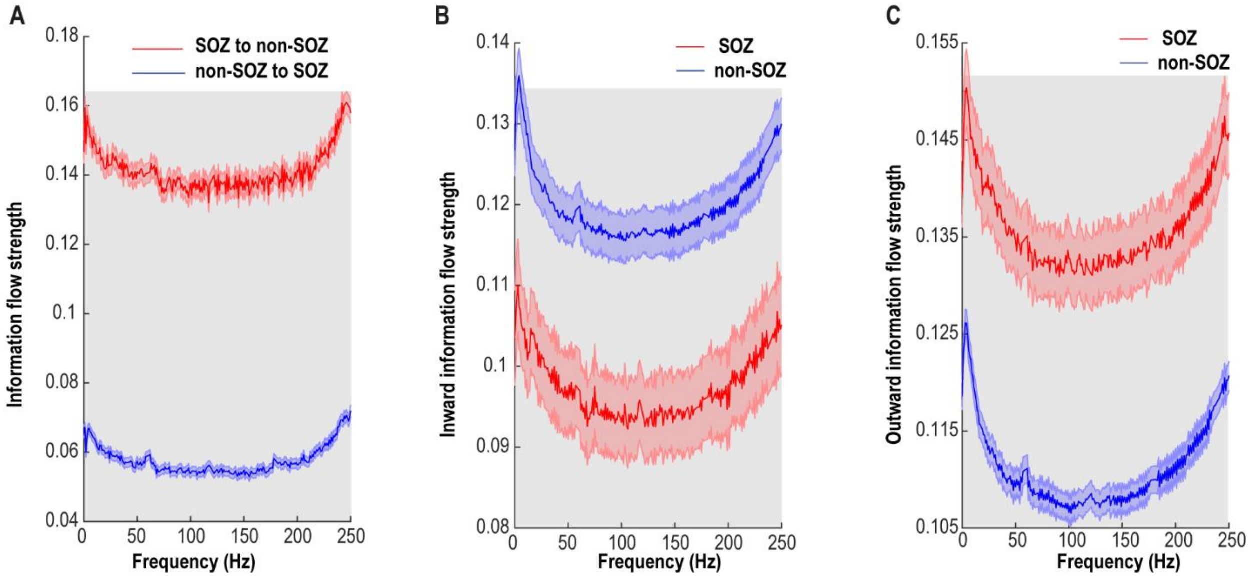

Figure S1 Within-frequency information flow during the ictal period. (A) Mean bidirectional information between SOZ and non-SOZ across all electrode pairs and patients. (B) Inward (receiving) information flow strength in SOZ and non-SOZ. (C) Outward (sending) information flow strength in SOZ and non-SOZ. Data are shown in mean and standard error. The shaded gray area indicates significant differences at the *p*=0.01 level after multiple comparisons.

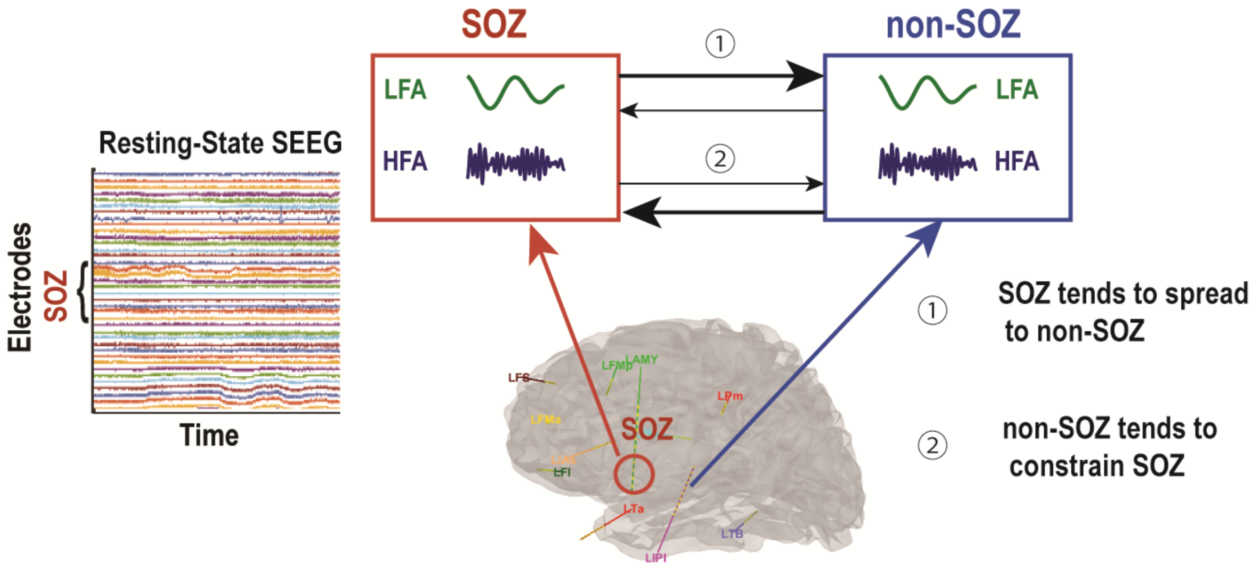

Figure S2 Hypothesized antagonism of information flows at multiple frequencies between SOZ and non-SOZ underlying epilepsy resting state. There are two possible scenarios: 1) information present in the SOZ tends to spread to non-SOZ; 2) non-SOZ activity tends to inhibit information transfer from the SOZ. The information flows could be transferred by low-frequency activity (LFA, <30 Hz), high-frequency activity (HFA, >30 Hz), and their interactions.

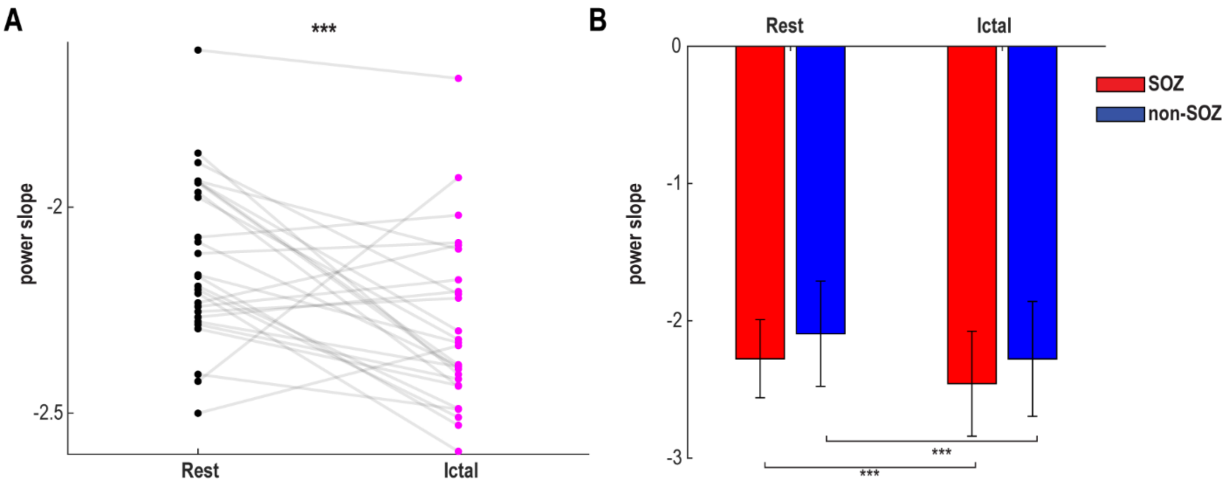

Figure S3 Power slope during the resting-state period and ictal period. There are significant differences in power slope between resting-state period and ictal period. Error bar represents standard deviation. *** $p<0.001$;

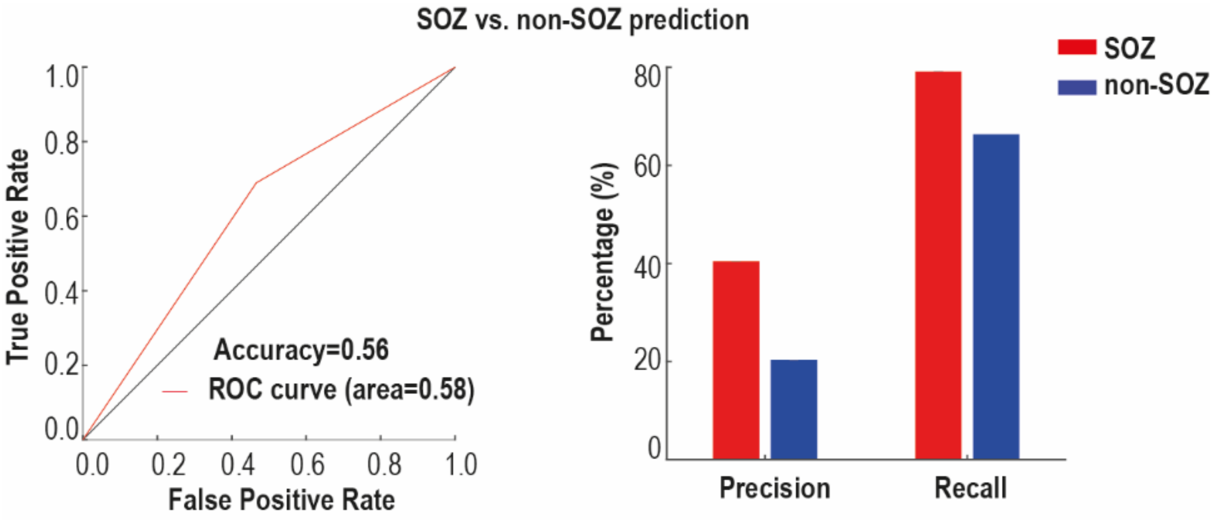

Figure S4 Performance of SOZ predictions at the individual level using 1/f power slope as features. Receiver-operating characteristic (ROC) curves show the true-positive and false-positive rates in predicting SOZ vs. non-SOZ. The area under the curve (AUC) is 0.58. Precision = True Positive / (True Positive + False Positive); Recall= True Positive / (True Positive + False Negative).

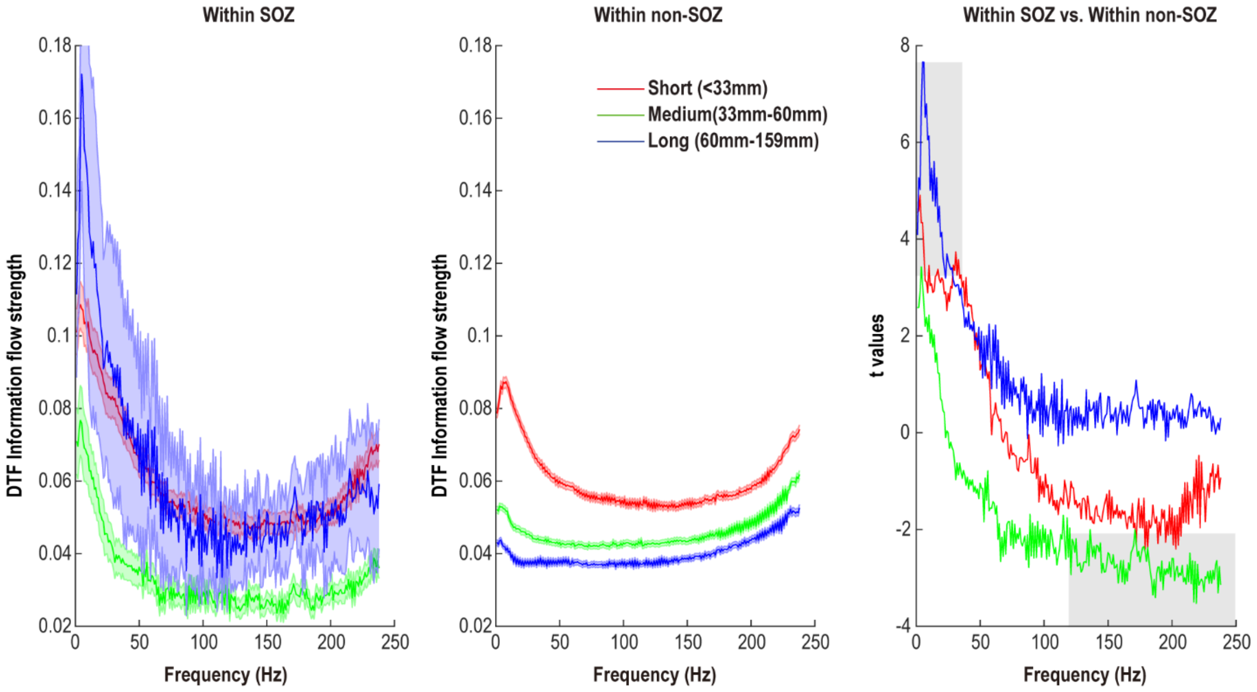

Figure S5 Within-frequency directional information flow as a function of distance during the resting-state period. Left panel: Mean DTF information flow of all electrode pairs within SOZ in distance-range quartiles. Middle panel: Mean DTF information flow of all electrode pairs within non-SOZ in distance-range quartiles. Right panel: Statistical difference between DTF information flow within SOZ and DTF information flow within non-SOZ in distance-range quartiles. Significant area at the p=0.05 level after FDR correction is marked in shadow. Data are shown in mean and standard error.

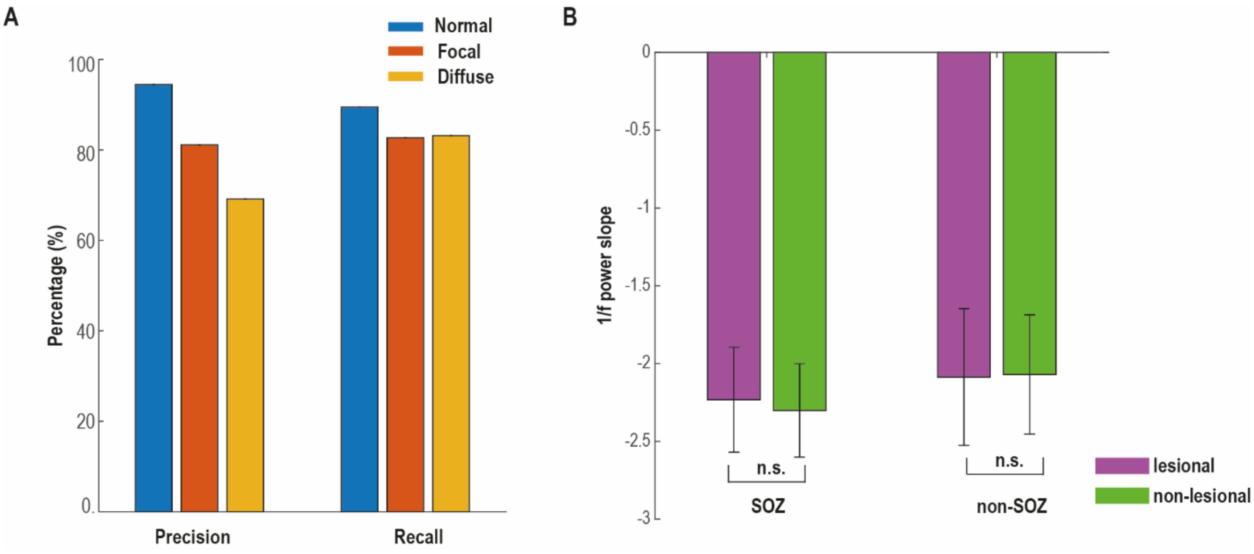

Figure S6 Metric variations corresponding to MRI findings. (A) SOZ prediction performance in correspondence to the MRI type. The SOZ prediction achieved best performance in patients without MR visible lesions. The SOZ prediction in patients with focal lesions performed better than in patients with diffuse MRI visible lesions. (B) 1/f power slope in patients with and without lesions. n.s.= not significant.

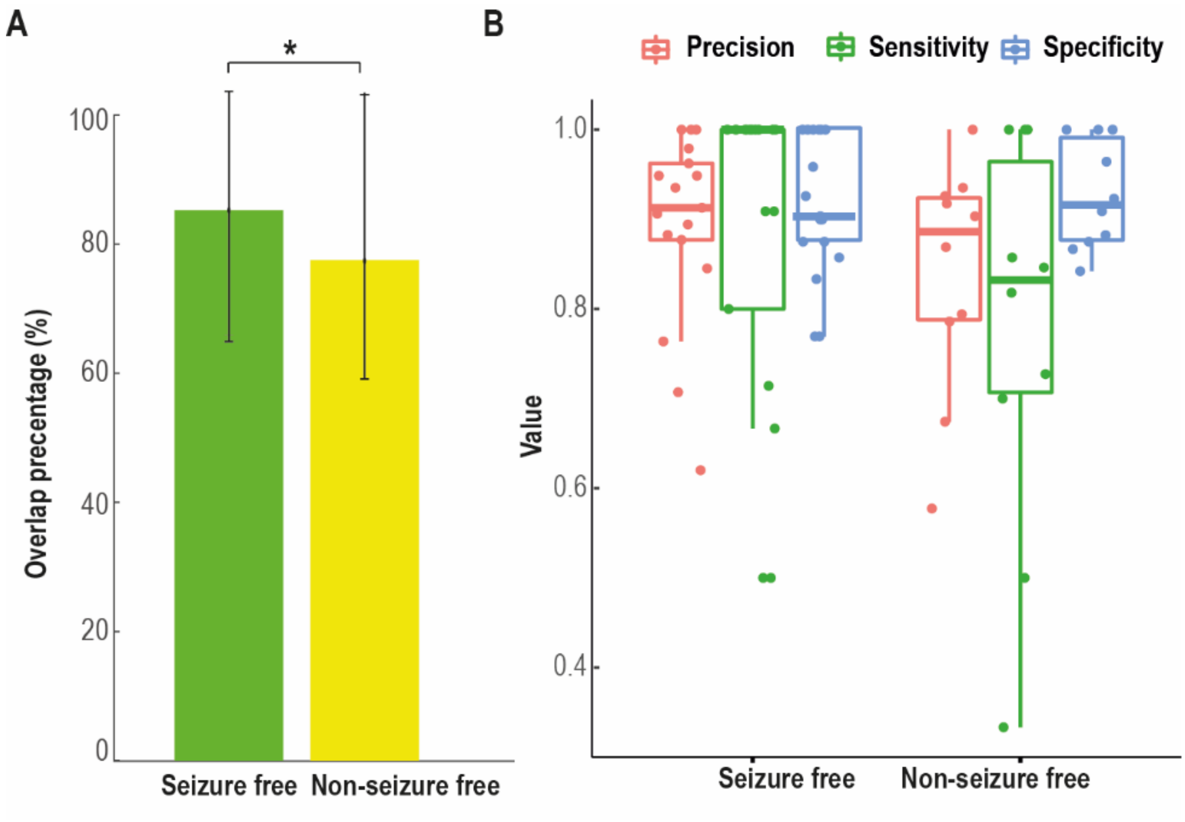

Figure S7 SOZ prediction performance in relation to seizure outcome. (A) The overlap between the "proposed model predicted SOZ" and the clinically determined SOZ in seizure-free and in non-seizure free groups. (B) Performance of concordance in detecting the SOZ (sensitivity, specificity, precision) at the individual patient level. *$p<0.05$

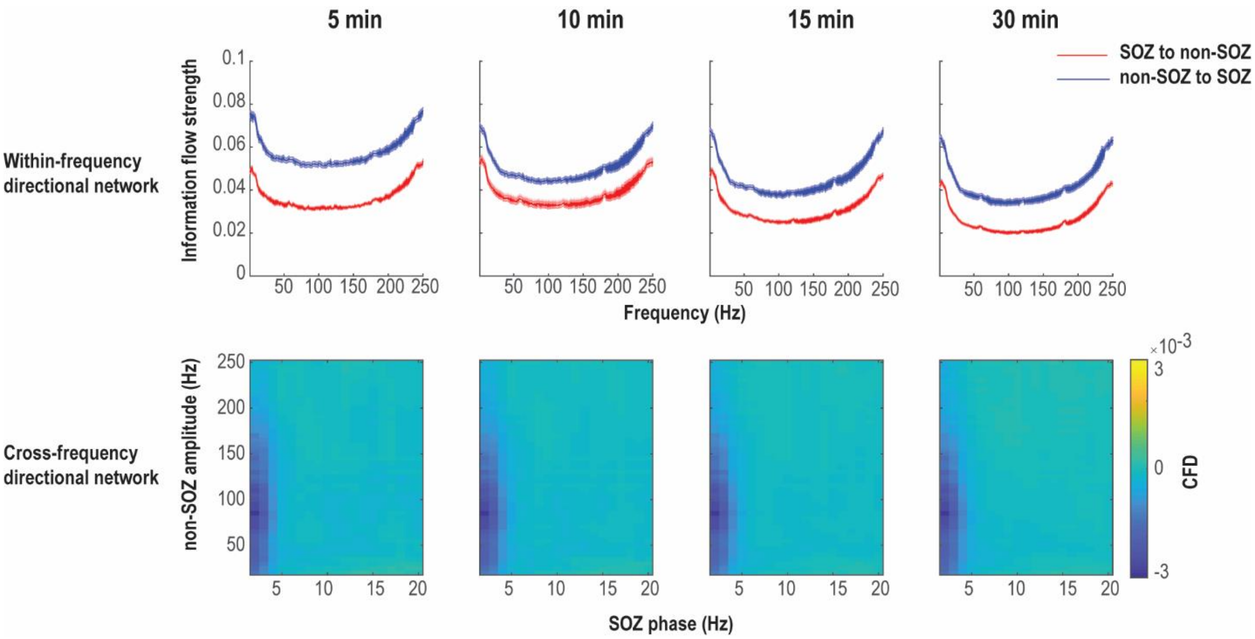

Figure S8 Control analysis of different durations in within-frequency directional network and cross-frequency directional network analysis during the resting state period. The directed functional connectivity is averaged over all SOZ and non-SOZ electrode pairs in all patients. The overall patterns are similar in 5 min, 10 min, 15 min and 30 min of resting-state.

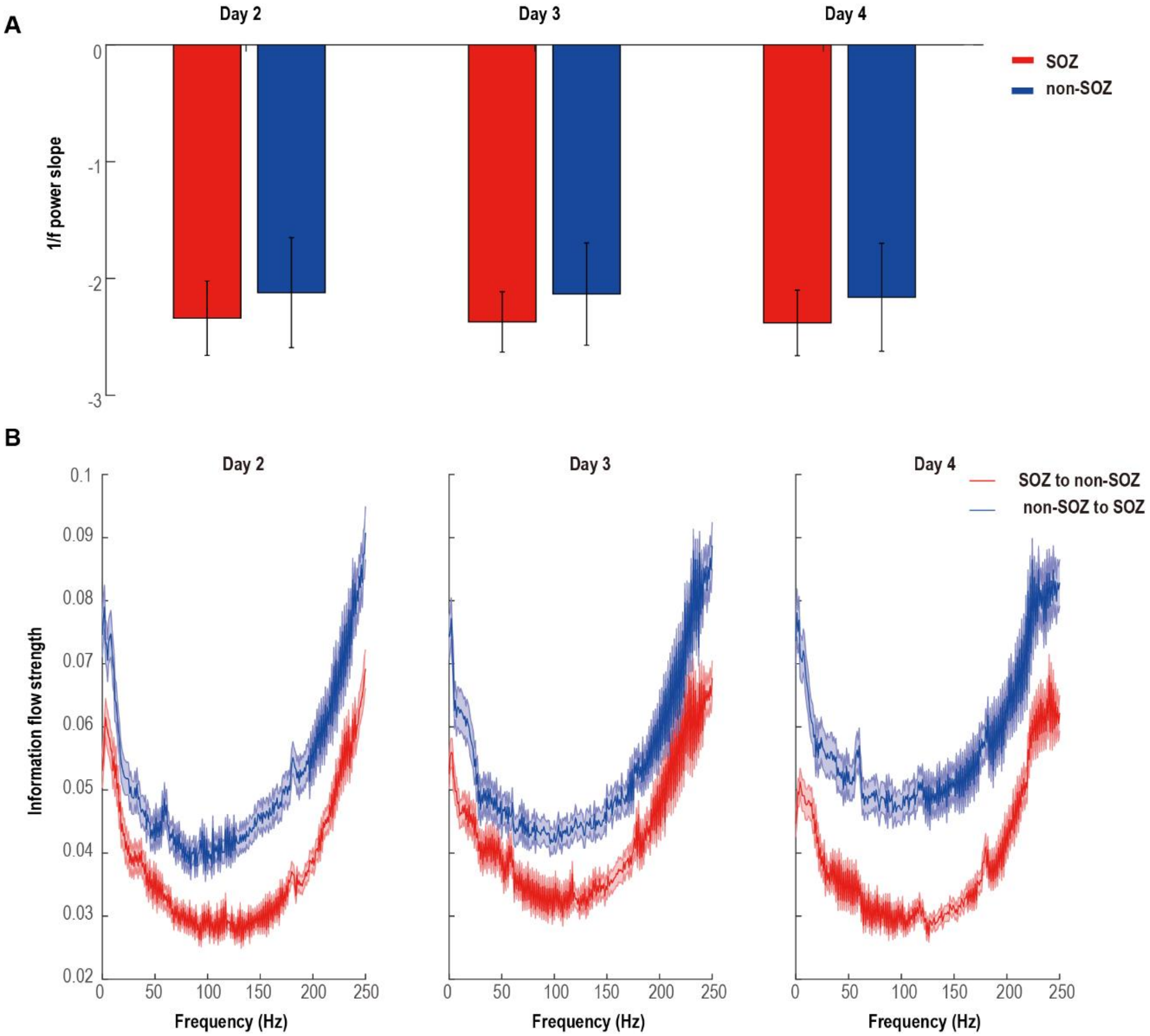

Figure S9 Stability of 1/f power slope and connectivity during the resting state in five selected patients over three different days. (A) 1/f power slope averaged over all SOZ and non-SOZ electrodes. (B) Within-frequency information flow averaged over all SOZ and non-SOZ electrode pairs. Results are grand averaged over 5 patients, and data are shown in mean and standard error.

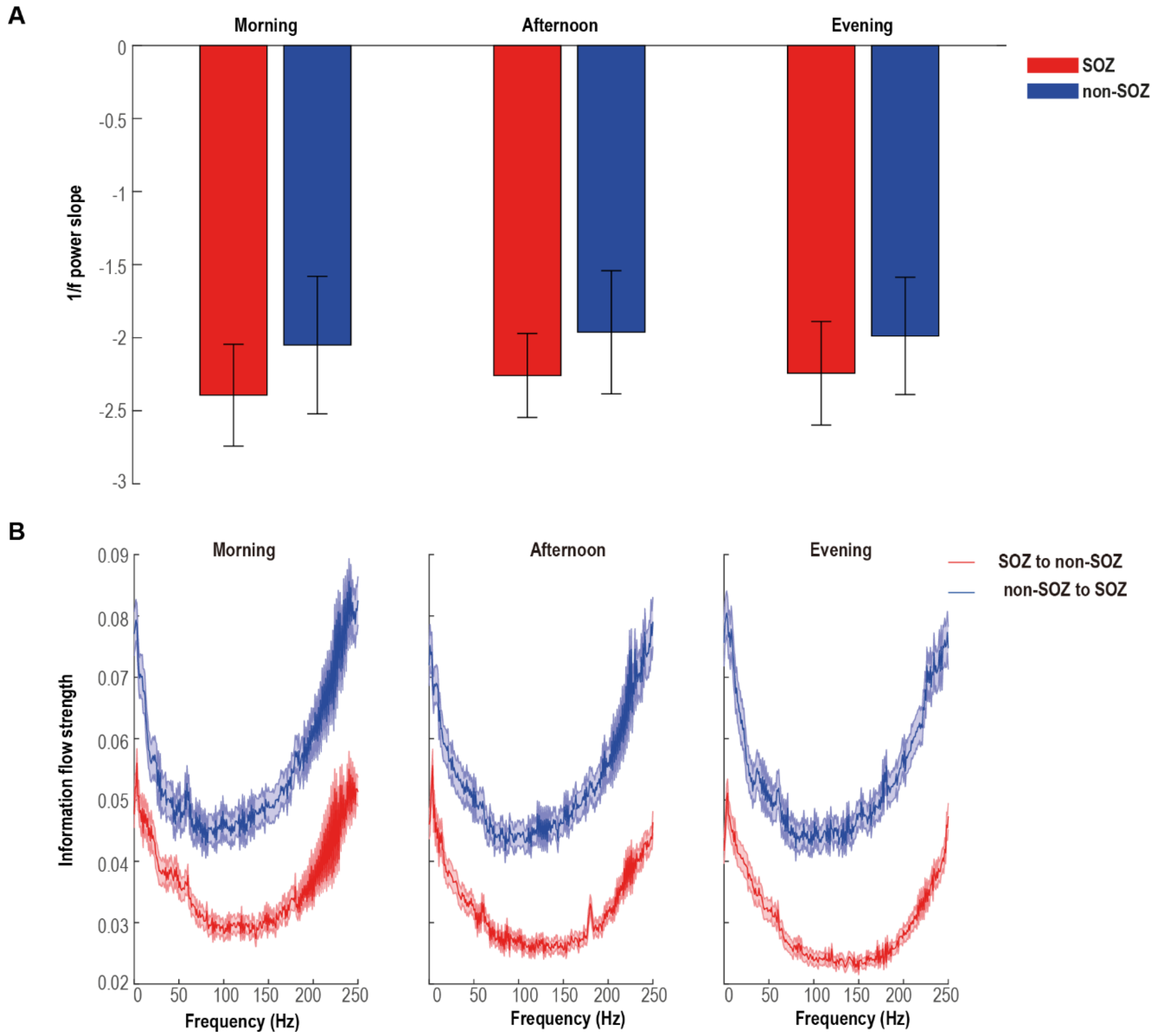

Figure S10 Stability of 1/f power slope and functionao connectivity during the resting state in five selected patients over three different time periods (Morning/Afternoon/Evening). (A) 1/f power slope averaged over all SOZ and non-SOZ electrodes. (B) Within-frequency information flow averaged over all SOZ and non-SOZ electrode pairs. Results are grand averaged over 5 patients, and data are shown in mean and standard error.

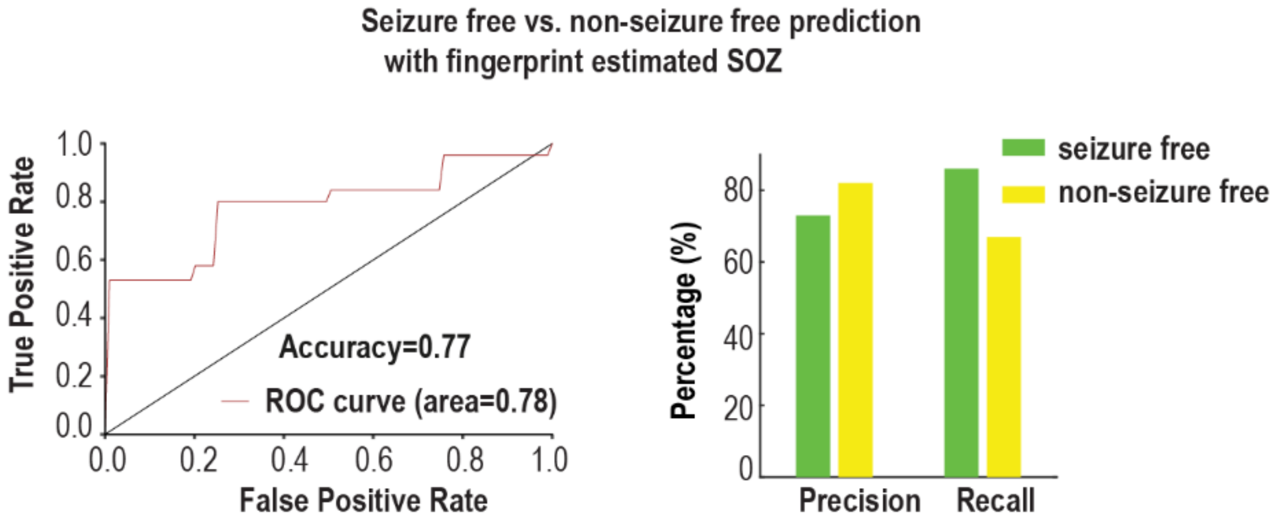

Figure S11 Outcome prediction with fingerprint estimated SOZ, where only 10-min resting state SEEG data were used. Receiver-operating characteristic (ROC) curves show the true-positive and false-positive rates in predicting seizure free vs. non-seizure free outcome. The area under the curve (AUC) is 078. The percentage evaluates the performance of outcome prediction. Precision = True Positive / (True Positive + False Positive); Recall= True Positive / (True Positive + False Negative).

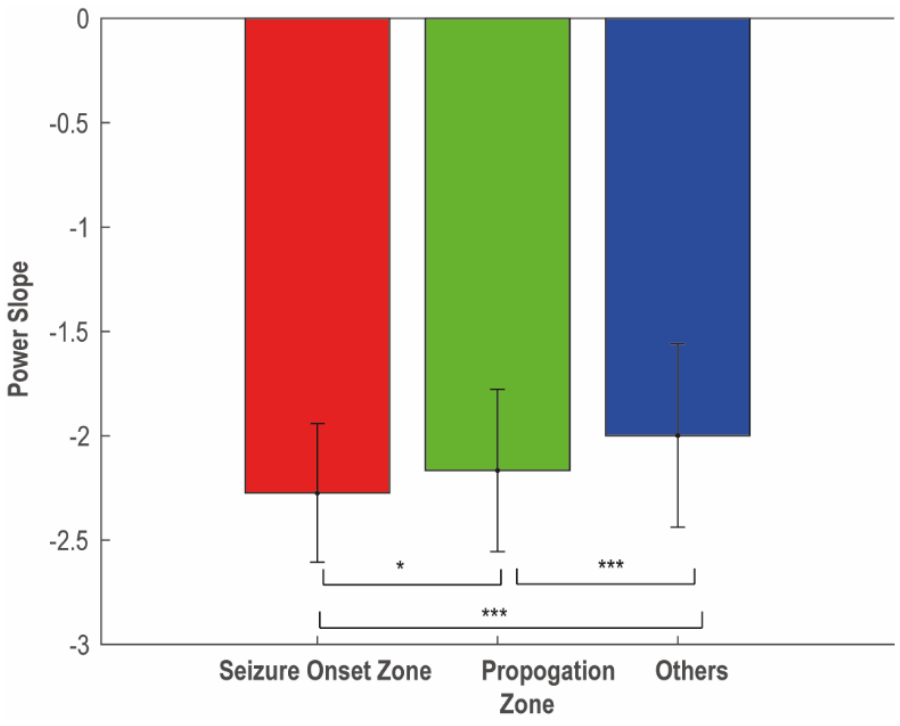

Figure S12 E:I ratio inferred by 1/f power slope in seizure onset zone, propagation zone and other zones. * p<0.05, *** p<0.001.

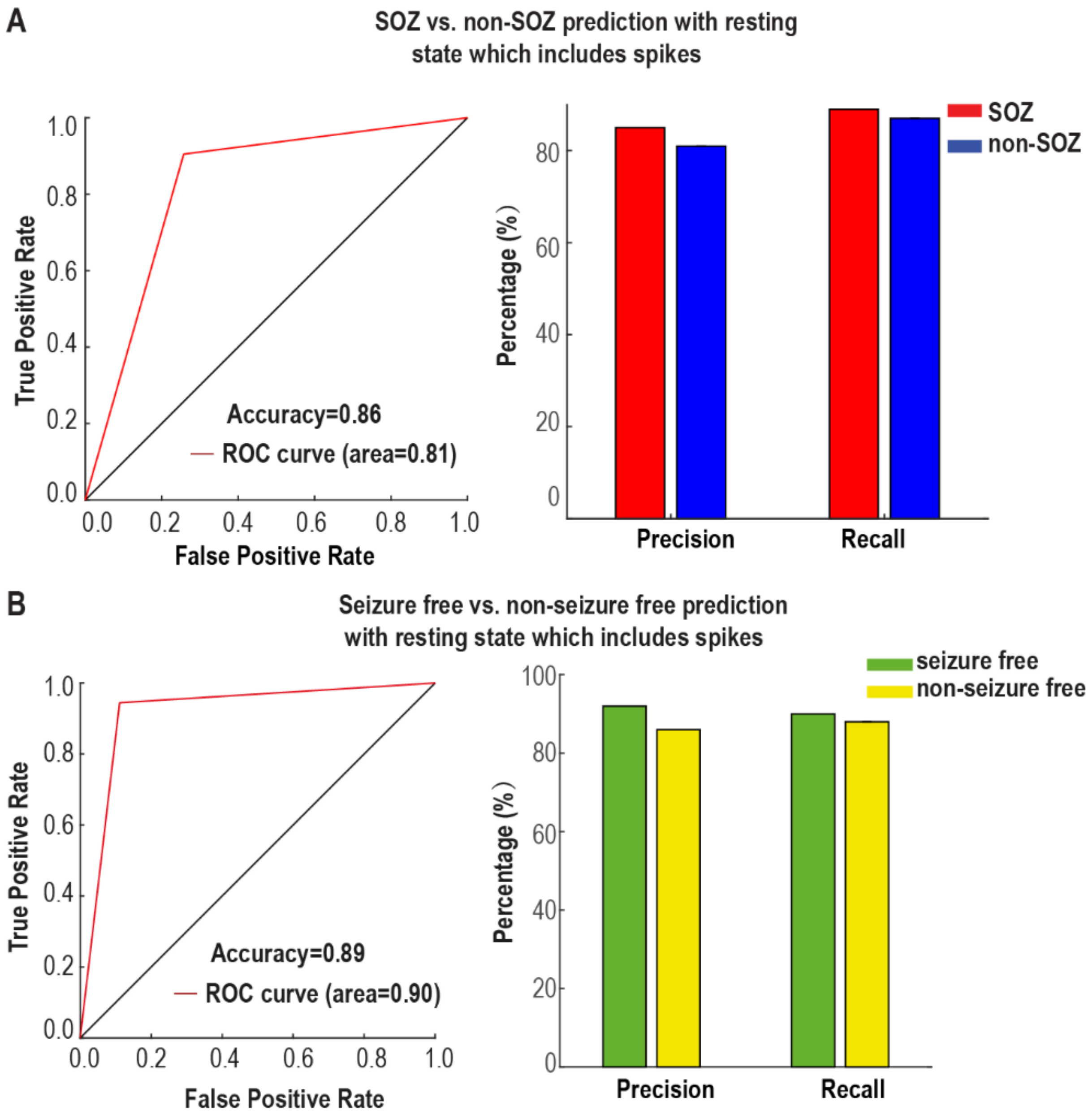

Figure S13 SOZ and seizure outcome prediction from short-duration resting-state SEEG recordings which includes interictal spikes. (A) SOZ prediction. (B) Seizure outcome prediction. Receiver-operating characteristic (ROC) curves show the true-positive and false-positive rates in predicting seizure free vs. non-seizure free outcome. Precision = True Positive / (True Positive + False Positive); Recall= True Positive / (True Positive + False Negative).

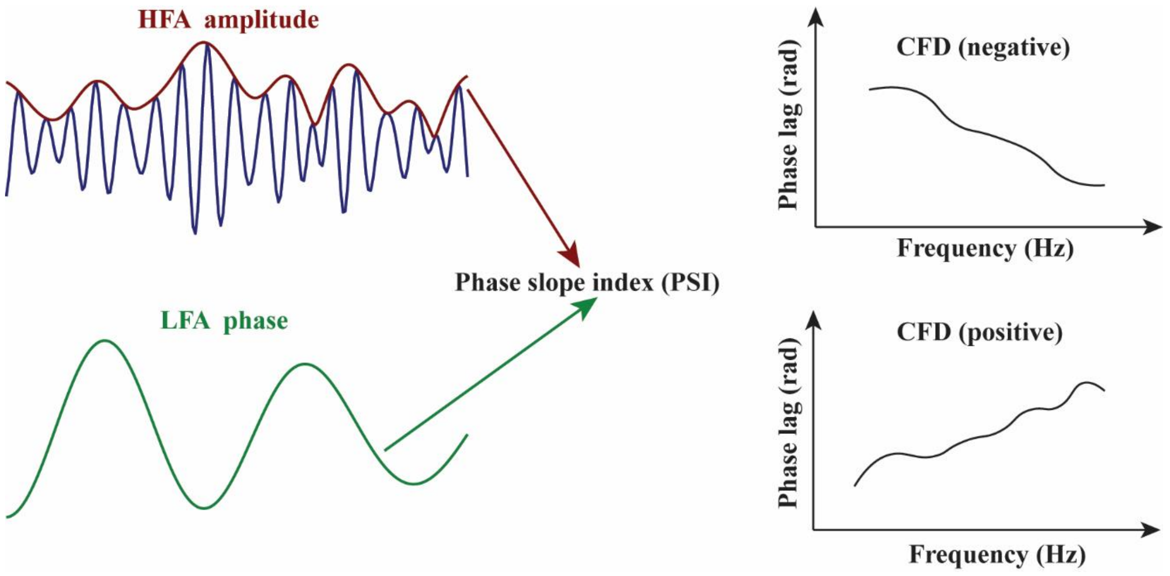

Figure S14 Illustration of cross-frequency directionality (CFD). CFD could evaluate directional interactions between different frequencies by computing the phase-slope index (PSI) between the LFA phase and the HFA amplitude. PSI is a robust method to quantify directionality because it allows one to infer whether one signal leads or lags a second signal by considering the slope of phase differences in a pre-specified frequency range. Therefore, the positive CFD indicates that the LFA phase drives the amplitude of HFA and vice versa for the negative CFD.
